# Validation of the Ankylosing Spondylitis Quality of Life assessment tool in patients with non-radiographic axial spondyloarthritis

**DOI:** 10.1007/s11136-020-02686-0

**Published:** 2020-10-31

**Authors:** Bengt Hoepken, Daniel Serrano, Kristina Harris, Mark C. Hwang, John Reveille

**Affiliations:** 1 UCB Pharma, Alfred-Nobel-Strasse 10, 40789 Monheim am Rhein, Germany; 2 Pharmerit International, Bethesda, MD USA; 3 UCB Pharma, Hong Kong, China; 4 McGovern Medical School, The University of Texas Health Science Center at Houston, Houston, TX USA

**Keywords:** Ankylosing Spondylitis, ASQoL, Axial spondyloarthritis, Non-radiographic axial spondyloarthritis, Patient-reported outcomes

## Abstract

**Purpose:**

To evaluate the psychometric performance of the Ankylosing Spondylitis Quality of Life (ASQoL) scale in patients with non-radiographic axial spondyloarthritis (nr-axSpA) to assess its appropriateness as an outcome measure in future clinical studies.

**Methods:**

Patients with active axSpA from a Phase III, randomized, double-blind, placebo-controlled trial (RAPID-axSpA, NCT01087762) were included (*N* = 325). Modified New York (mNY) classification criteria were used to classify patients as having ankylosing spondylitis or nr-axSpA; those with nr-axSpA were further categorized based on objective signs of inflammation. Psychometric properties of the ASQoL were assessed/documented using a mixture of modern psychometric methods and classical test theory methods. These included exploratory factor analysis and item response theory models to assess the domain structure, test the utility of a single domain relative to subdomains, assess bias, and generate statistics to guide an empirical scoring algorithm. The reliability and validity of scores were evaluated via internal consistency, test–retest reliability, concurrent validity, and known-groups validity. Score responsiveness was assessed via anchor-based clinically meaningful change, supplemented with empirical cumulative distribution function visualizations.

**Results:**

The ASQoL data were defined by four domains. However, a four-domain solution was found to be inferior to a bifactor solution in which the four domains were included within a total domain. Scoring statistics supported a unit-weighted total score. Within the nr-axSpA population with objective signs of inflammation, the ASQoL mean score had adequate reliability, validity, and ability to detect clinically meaningful change.

**Conclusions:**

Our findings suggest that the ASQoL is an appropriate outcome measure in interventional clinical trials in patients with nr-axSpA.

**Electronic supplementary material:**

The online version of this article (10.1007/s11136-020-02686-0) contains supplementary material, which is available to authorized users.

## Introduction

Axial spondyloarthritis (axSpA) is a chronic inflammatory disease predominantly affecting the axial skeleton [sacroiliac (SI) joints and spine] that includes ankylosing spondylitis (AS) and non-radiographic axial spondyloarthritis (nr-axSpA). The main difference between AS and nr-axSpA is the presence of clear structural changes in SI joints on pelvic radiographs in AS patients and absence or low evidence of this radiographic damage in nr-axSpA. Both AS and nr-axSpA patients often show active inflammation on magnetic resonance imaging (MRI) of the SI joints and spine which, over time, may evolve into chronic lesions (erosions, fat lesions, sclerosis, and ankylosis) leading to radiographically detectable structural damage [1–6]. Slight differences observed between these subtypes may represent different stages of the disease and also different disease courses. The prevalence of axSpA in patients with and without radiographic changes has been shown to be similar, both subtypes being equally relevant [7]. Onset of symptoms is typically in late adolescence or early adulthood [8], although diagnosis is often delayed, taking around 7 years to be confirmed, and may take significantly longer in females than in males [9, 10].

Regardless of the presence or absence of structural damage, both AS and nr-axSpA patient populations display the characteristic symptoms of axSpA [1–6], including pain, stiffness and impaired physical function [4, 11, 12]. Patients may also be affected by other disease manifestations such as peripheral arthritis, enthesitis, acute anterior uveitis, psoriasis and inflammatory bowel disease, as well as having an increased risk of osteoporosis, atherosclerotic events, and cardiovascular problems [13]. The impact of axSpA extends beyond the physical symptoms, affecting patients’ ability to work as well as being associated with high levels of fatigue and psychological distress [14]. Given disease onset typically occurs during early adulthood, its effects on patient quality of life are of considerable duration [14].

The burden of disease in terms of effects on health-related quality of life is similar in both AS and nr-axSpA [15]. Two studies conducted in Scandinavia [16, 17] reported that, compared to the general population, patients with axSpA had significantly lower scores in all eight dimensions of the generic short-form 36 assessment (all *p* < 0.001), with greater impairments seen in physical domain scores. A survey of 2846 patients across 13 European countries reported that axSpA was directly responsible for difficulty finding and keeping employment in 74% of patients, with 62% experiencing psychological distress [18].

The Ankylosing Spondylitis Quality of Life (ASQoL) scale is an 18-item dichotomous patient-reported outcome (PRO) measure allowing calculation of a total score ranging from 0 to 18, and was developed to assess the impact of interventions for AS on quality of life [19]. There is no such PRO measure designed specifically for use in patients with nr-axSpA.

The objectives of this study were to re-evaluate the domain structure and scoring of the ASQoL and to demonstrate the reliability, validity and responsiveness of the ASQoL in patients with nr-axSpA and objective signs of inflammation.

Prior to this, patient interviews were conducted, which confirmed the concepts measured by the ASQoL were relevant to patients with nr-axSpA and provided the first step in assessing the validity of the ASQoL in this patient population [20].

## Methods

### Study design and participants

Data from patients with active axSpA enrolled in a 24-week Phase III, multicenter, randomized, double-blind, placebo-controlled trial (RAPID-axSpA, NCT01087762) [21] were used in this analysis. Eligible patients were ≥18 years with a documented diagnosis of adult-onset axSpA of at least 3 months’ duration as defined by the ASAS axSpA classification criteria, a Bath Ankylosing Spondylitis Disease Activity Index (BASDAI) [22] score ≥4, spinal pain ≥4 on a 0–10 numeric rating scale, C-reactive protein (CRP) greater than the upper limit of normal and/or evidence within three months of screening of sacroiliitis on magnetic resonance imaging (MRI) or X-ray as defined by Assessment of SpondyloArthritis international Society (ASAS)/Outcome Measures in Rheumatology (OMERACT) scoring [23]. All pelvic radiographs and MRI scans were assessed and confirmed by two central readers and, if necessary, an adjudicator. Patients were also required to be intolerant of non-steroidal anti-inflammatory drugs (NSAIDs) or have had an inadequate response to at least one NSAID after at least 30 days of treatment or to two NSAIDs after at least two weeks of treatment with each. The RAPID-axSpA study, from which the patient data used in this study were derived, had been approved by the independent ethics committee or institutional review board at participating sites, and written informed consent obtained from all patients.

Patients were classified as having AS [fulfilling ASAS axSpA classification criteria and modified New York (mNY) classification criteria [6]] or nr-axSpA (fulfilling ASAS axSpA classification criteria but not mNY classification criteria). Patients with nr-axSpA were further classified using the more stringent objective signs of inflammation criteria [24], defined as a Spondyloarthritis Research Consortium of Canada [SPARCC] [25] score ≥2 of MRI scans of the SI joint and/or serum C-reactive protein levels exceeding the upper limit of normal.

The following eight PROs were used in various stages of this evaluation of the ASQoL psychometric properties:

- Patient Global Impression of Change (PGIC) [26].
- Bath Ankylosing Spondylitis Disease Activity Index (BASDAI) [22].
- Bath Ankylosing Spondylitis Functional Index (BASFI) [27].
- Patient Global Assessment of Disease Activity (PtGADA) [28].
- Physician Global Assessment of Disease Activity (PhGADA) [29].
- Total and nocturnal spinal pain numeric rating scales.
- Ankylosing Spondylitis Disease Activity Score (ASDAS) [30].
- Short-Form 36 Health Survey version 2 (SF-36v2) [31].

### Statistical analysis

Data were described using standard descriptive statistics to characterize the overall patient population and subpopulations. Response pattern evaluations were also performed to assess inter-item tetrachoric correlations. Cross-sectional analyses, including the modern psychometric methods (MPMs), were conducted on baseline data. This approach enables evaluation of the psychometric properties of the ASQoL prior to any experimental and/or pharmacogenic interventions that could alter the underlying disease evaluated by the ASQoL. These analyses were complemented by sensitivity cross-sectional analyses at later time points.

The total axSpA intent-to-treat patient population was used for all MPMs, with further analyses performed on subpopulations (patients diagnosed with nr-axSpA overall as well as in the subgroups with and without objective signs of inflammation). Readers interested in considerations related to sample size and estimation of these models are directed to the discussion wherein a section is dedicated to the finite sample properties of estimability and bias.

MPMs employed a combination of full information item exploratory factor analysis (EFA) and item response theory (IRT) [32]. These methods generated evidence guiding domain specification, item performance evaluation, assessments of item bias, and scoring.

Following current best practice, the number of domains (factors) was determined from model fit indices [33]. These included the C2 *χ*^2^ test of absolute fit [33] and the C2-based root mean squared error of approximation (RMSEA) goodness of fit test [34], and standard metrics were used for interpreting the estimates [35]. Interpretability of the final domain solutions was achieved through oblique Quartimax rotation.

Four alternative confirmatory IRT structures were assessed to evaluate item performance, bias, and empirically guide scoring. IRT models considered included a two-parameter logistic (2PL) model [36, 37], a Rasch analog of the 2PL model [38], a bifactor model [39], and a multidimensional item response theory (MIRT) model. In addition to the model fit assessment, item parameter quality and Chen’s local dependence statistic [40] were used to evaluate which IRT model best characterized the performance of the ASQoL items. Items with *χ*^2^ values exceeding 3 indicated potentially serious local dependence. Items with IRT slopes exceeding 4 were considered to be potentially unstable [41].

Differential item functioning (DIF) was used to assess whether ASQoL items functioned identically between axSpA subpopulations within the final ASQoL domain solution. This was performed using the Wald-2 DIF *χ*^2^ sweep procedure, with p-values adjusted for the false discovery rate using the Benjamini–Hochberg procedure [42]. Items identified as having significant DIF were further evaluated via a DIF severity assessment [43] to assess whether detected significant DIF would severely impact scores. For an item to be declared biased between axSpA subpopulations it had to demonstrate both significant and severe DIF.

The ASQoL score is presented as either the sum score (sum of score for each ASQoL item; scale of 0–18) or the mean score [sum score divided by 18 (total number of ASQoL items); scale 0–1]. For both, a lower score indicates better quality of life. As there was no item-level missing data, results were identical for any correlation-based analysis. The optimal scoring procedure was determined based on scoring statistics [44]. Four possible scores were considered and scoring statistics characterized the relative merits of each: unit-weighted (with each item given equal weighting) domain scores, unit-weighted total scores, and empirically weighted (each item weighted by its reliability) versions of domain and total scores.

Note that a Supplemental Web Appendix contains the complete tabular and graphical output of the modern psychometric results, and interested readers are directed there for additional evidence.

Internal consistency was assessed to characterize the performance of the ASQoL in addition to guiding scoring decisions. Four possible scores were evaluated via the *ω*-based statistics: unit-weighted domain scores, unit-weighted total scores, and empirically weighted versions of domain and total scores. Internal consistency was measured by McDonald’s *ω* statistic and the corresponding bifactor analog, *ω*_*H*_ [44]. These statistics are the least biased internal consistency estimators [42]. Subdomain scores would be supported if ω exceeded *ω*_*H*_, and total scores would be supported if *ω*_*H*_ exceeded ω. Further, as the *ω*_*H*_/*ω* ratio approaches 1, a total domain is favored. Low values (<0.7) on both *ω* and *ω*_*H*_ indicate a need for empirically weighted scores.

ASQoL score performance was evaluated in terms of the test characteristic curve (TCC) and the precision of score measurement via the test information function (TIF). Additional assessments included estimates of test–retest reliability, validity, ability to detect change (responsiveness) and meaningful within-patient change (MWPC).

Test–retest reliability of the ASQoL responses was estimated correlating Baseline with Week 12 and Week 24 follow-up data. Test–retest reliability was estimated in a group of stable patients, defined as those patients with no change in PGIC, PtGADA (defined as a change in scores between ±1 point), or PhGADA (defined as a change in score between ±15 points). The analysis was based on the two-way random intraclass correlation coefficient (ICC[2, 1]) [45] with estimates of at least 0.7 prespecified as indicating acceptable reproducibility of scores. Given the length of the retest interval and the fact that the retest interval spanned the interventional period of the randomized trial, the evidence presented for test–retest reliability could better be described as long-term stability. To remain consistent with regulatory review and interaction, in this manuscript we retain the description of this evidence as test–retest reliability.

Concurrent validity (both convergent and divergent) was estimated at baseline via Spearman correlations within the nr-axSpA population with objective signs of inflammation. Sensitivity analyses were conducted at Weeks 12 and 24. Convergent validity estimates were obtained by correlating ASQoL total scores and those of the BASDAI, BASFI, PtGADA, PhGADA, total and nocturnal spinal pain numeric rating scales, and the ASDAS composite score. Divergent validity estimates were obtained by correlating the ASQoL total scores with the physical functioning and physical component scores of the SF-36v2. The ability of the tests to detect change was also determined from the Spearman correlation coefficient for change in scores from Baseline to Week 12 and to Week 24 for ASQoL versus other PRO measures. In all cases correlations ≥|0.4| met the prespecified criterion for acceptable validity.

Known-groups validity evidence was generated at Baseline, Week 12 and Week 24. Scores from the PhGADA and ASDAS were dichotomized (median split and cut at 2.1, respectively) to define known groups. The mean differences in ASQoL score between the known groups for each measure were analyzed using analysis of variance (ANOVA).

MWPC was estimated by both distribution and anchor-based methods. Given regulatory emphasis on anchor-based methods, only anchor-based evidence is reported. The anchor-based method for MWPC estimation was based on patients whose change in ASQoL score between Baseline and Week 12 and Week 24 was equal to or greater than the estimated median change in ASQoL score in patients with a PGIC of 6 (moderate improvement) or 5 (minimal improvement). In addition, the MWPC point estimate was validated via empirical cumulative distribution functions (eCDFs) and 95% Clopper-Pearson confidence bands for change in ASQoL score from Baseline, stratified by PGIC strata (no change, minimal improvement, moderate improvement, marked improvement).

All analyses used observed case data only; no imputation of missing values was undertaken.

MPMs were conducted using FlexMIRT version 3.5 (Vector Psychometric Group). All other analyses were performed using a combination of Statistical Analysis Software version 9.4 (SAS^®^ Institute Inc., Cary, NC) and R statistical software version 3.4.3 (R Development Core Team).

## Results

All 325 patients from the Phase III study were included in the analysis; based on the ASAS axSpA classification criteria and fulfillment of the mNY classification criteria (using central assessment of X-rays) or not, 178 (54.8%) patients had AS and 147 (45.2%) had nr-axSpA. Of patients with nr-axSpA 67 (20.6%) had objective signs of inflammation (SPARCC ≥2 and/or elevated C-reactive protein).

Patient demographics and baseline disease characteristics are shown in Table 1 for all patients and for the patient subgroups. Overall, the majority (61.5%) of patients were male, had a mean (SD) age of 39.6 (11.9) years and a mean (SD) disease duration of 10.4 (9.5) years. Patients with nr-axSpA and objective signs of inflammation had shorter disease duration and were more likely to be female.

**Table 1:** Patient demographics and baseline disease characteristics

	All patients (*N* = 325)	nr-axSpA (*n* = 147)	AS (*n* = 178)	nr-axSpA with objective signs of inflammation (*n* = 67)	nr-axSpA without objective signs of inflammation plus AS (*n* = 258)^a^
Age (years)					
Mean (SD)	39.6 (11.9)	37.4 (11.8)	41.5 (11.6)	40.4 (12.8)	39.4 (11.6)
Range	19–78	19–78	19–68	19–78	19–68
Sex, *n* (%)					
Male	200 (61.5)	71 (48.3)	129 (72.5)	27 (40.3)	173 (67.0)
Disease duration (years)					
Mean (SD)	10.4 (9.5)	8.6 (8.6)	11.9 (9.9)	5.1 (5.7)	7.1 (7.8)
Range	0–51.0	0–42.0	0–51.0	0–24.2	0.1–37.9
Age at diagnosis (years)					
Mean (SD)	33.0 (11.1)	32.6 (11.6)	33.3 (10.6)	35.4 (13.0)	32.3 (10.5)
Range	6–78	10–78	6–66	16–78	6–66

^a^Patient subgroup included the 80 patients with nr-axSpA who did not have objective signs of inflammation together with the 178 patients with AS

*AS* ankylosing spondylitis,* axSpA* axial spondyloarthritis, *nr* non-radiographic, *SD* standard deviation

### Domain specification, item performance, and scoring

These assessments were conducted in the total patient population (*N* = 325). Response pattern evaluation showed that all items in the ASQoL were well endorsed (>20%, a common boundary for insufficient endorsement [46]). The lowest endorsement rates were 22.2% for difficulties in washing hair (ASQoL item 16) and 37.0% for being unable to do chores (ASQoL item 11); all other items had endorsement values of >54%. No items crossed the common over-endorsement criterion of 90%; the strongest endorsements were 89.6% for morning delay, 84.9% for being easily tired, and 83.1% for being always in pain (ASQoL items 10, 12, and 14, respectively).

Results from the EFA showed that the ASQoL item response associations could be explained by four domains as this achieved an optimal fit with the fewest extracted factors. This solution satisfied the C2 *χ*^2^ test of absolute fit (*p* = 0.27) as did the RMSEA and corresponding 90% CI (0.02 [0.00, 0.04]). Oblique quartimax rotation of this solution yielded a logical mixture of symptoms and impacts relating to the ASQoL items. Observed domains characterized: activities of daily living and pain; sleep disturbance and activity limitation; emotion; and fatigue. These domains were tested in the MIRT and bifactor IRT models described next.

The four confirmatory IRT structures could not be differentiated based on the *χ*^2^ test of absolute fit (all *p* < 0.005). However, both of the unidimensional models were rejected as they showed evidence of strong local dependence. Although the bifactor model showed a large local dependence estimate between ASQoL items 13 (frustrated) and 7 (always fatigued), it was the only model that satisfied the goodness of fit test (RMSEA [90% CI]: 0.03 [0.000, 0.049]). The bifactor model demonstrated acceptable IRT slope stability, with only ASQoL items 4 (struggle to do chores) and 12 (easily fatigued) having slopes >3 (Table 2). Taking all of these findings into consideration, the bifactor model was selected as the final model from which test and score properties would be evaluated.

**Table 2:** IRT slopes for the MIRT and bifactor models

ASQoL item	ASQoL item short description	Domain	MIRT slope	Bifactor slope
1	Limits	Activities of daily living and pain	1.95	1.80
3	Dressing	Activities of daily living and pain	1.21	0.92
4	Struggle chores	Activities of daily living and pain	3.71	5.22
9	Unbearable pain	Activities of daily living and pain	1.67	1.61
10	Morning delay	Activities of daily living and pain	1.45	1.43
11	Unable chores	Activities of daily living and pain	2.77	2.32
14	Always pain	Activities of daily living and pain	1.15	1.17
16	Hair	Activities of daily living and pain	1.39	1.16
5	Sleep disturbance	Sleep disturbance and activity limitation	1.14	1.10
6	Unable activities	Sleep disturbance and activity limitation	2.81	2.08
7	Always fatigued	Fatigue	2.32	1.79
8	Rest	Fatigue	2.07	1.91
12	Easily fatigued	Fatigue	4.64	5.53
2	Crying	Emotion	1.40	1.15
13	Frustrated	Emotion	2.31	1.88
15	Miss out	Emotion	2.47	2.51
17	Depressed	Emotion	1.83	1.73
18	Disappoint	Emotion	1.59	1.45

Scoring statistics supported the use of a unit-weighted total score. The total score accounted for 90% of the explainable internal consistency and the subdomains accounted for only the remaining 10% of explainable internal consistency. The explained common variance associated with the total domain was 0.73, and the factor determinacy statistic (*H*) for the total domain was 0.95. These findings meet the standards set by Rodriguez, Reise, and Haviland [44] for favoring a unit-weighted total score. This provides empirical support for the developer’s scoring algorithm. All evidence presented hereafter pertains to a unit-weighted total score.

**Fig. 1:**
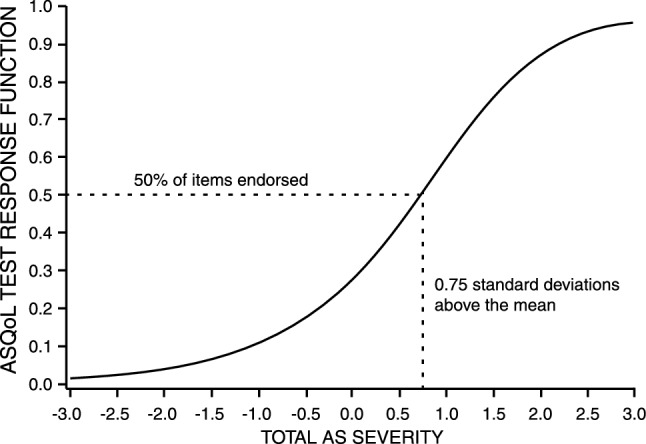
ASQoL test characteristic curve (TCC)

**Fig. 2:**
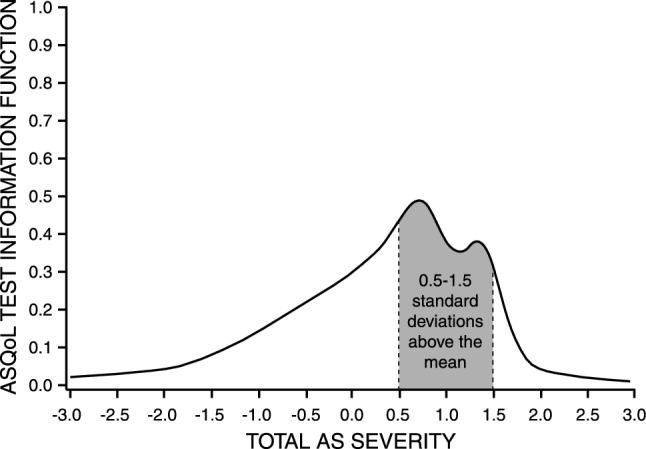
ASQoL test information function (TIF)

ASQoL mean score performance evaluated by the TCC demonstrated a strong score discrimination function, indicating that patients 0.75 standard deviations above the mean on total AS severity endorsed 50% of the ASQoL items (Fig. 1). The TIF demonstrated that score precision was maximized for patients located between 0.5 and 1.5 standard deviations above the mean on total AS severity (Fig. 2). Therefore, the ASQoL detected and characterized AS of moderate to moderately high severity, defined as scores falling between 0.5 and 1.5 standard deviations above the mean.

DIF testing identified significant DIF between patients with AS or nr-axSpA (classified by mNY criteria) for ASQoL items 7 (always fatigued; *p* = 0.0034) and 12 (easily fatigued; *p* = 0.0036). However, the DIF severity effect size and corresponding weighted area between curve (wABC) plots demonstrated that this significant DIF was not meaningful. None of the ASQoL items demonstrated significant DIF (*p* ≥ 0.0685) between nr-axSpA patient subgroups with or without objective signs of inflammation. Therefore, no meaningful DIF was detected in the ASQoL and there was no difference in its function between patients with AS or nr-axSpA irrespective of the method of classification. Note that when DIF analyses are based upon either a focal or reference group as small as the focal group in this application (*n* = 67), DIF can yield either increased Type I or Type II error rates, thus motivating the need for graphical scrutiny of DIF via the wABC.

### Assessment of score properties

These assessments were performed in the nr-axSpA with objective signs of inflammation patient population (*n *= 67) and compared with the population having AS or nr-axSpA without objective signs of inflammation (*n* = 258). Unit-weighted ASQoL mean scores, expressed as the proportion of the 18 items with a positive response, were 0.75 at Baseline (*n* = 67), 0.54 at Week 12 (*n* = 59), and 0.35 at Week 24 (*n* = 56).

The ASQoL total score had a high internal consistency (*ω*_*H*_ = 0.82) within this population. Test–retest reliability, anchored on no change in PGIC, PtGADA, or PhGADA score between Baseline and Week 12 or Week 24, gave ICC(2,1) estimates that exceeded the prespecified criterion of 0.7.

Concurrent validity of the ASQoL at Baseline exceeded the prespecified criterion for acceptable validity for all validators (*r* ≥ |0.50|), excepting PhGADA and ASDAS composite score (*r* = 0.24 and *r* = 0.34, respectively). Sensitivity analyses at Week 12 and Week 24 exceeded the strength of baseline findings with no validators failing to meet the prespecified criterion of acceptable validity.

While baseline known-groups validity did not detect significant differences, results at both Week 12 and Week 24 demonstrated that significantly worse mean ASQoL scores were seen for patients with ASDAS >2.1 or with PhGADA at or above the median value compared to patients in the known reference groups (*p* ≤ 0.002 and *p* ≤ 0.003, respectively). On average, patients with ASDAS >2.1 endorsed 32% more ASQoL items at Week 12 and 27% more items at Week 24 compared with patients with ASDAS <2.1. Similarly, patients with PhGADA scores at or above the median value endorsed approximately 29% and 26% more items at Weeks 12 and 24, respectively, compared with patients with PhGADA values below the median value.

The sensitivity of the ASQoL to detect changes in the PRO assessments between Baseline and Week 12 or Week 24 met or exceeded the standard criterion of *r* ≥ |0.4| for all assessments except for PtGADA at Week 12 (*r* = 0.36). Based on the PGIC moderate improvement anchor group, the point estimate (using ASQoL mean score) for MWPC at Week 12 was –0.22 and at Week 24 was –0.19, representing an improvement in approximately four of the 18 ASQoL items.

**Fig. 3:**
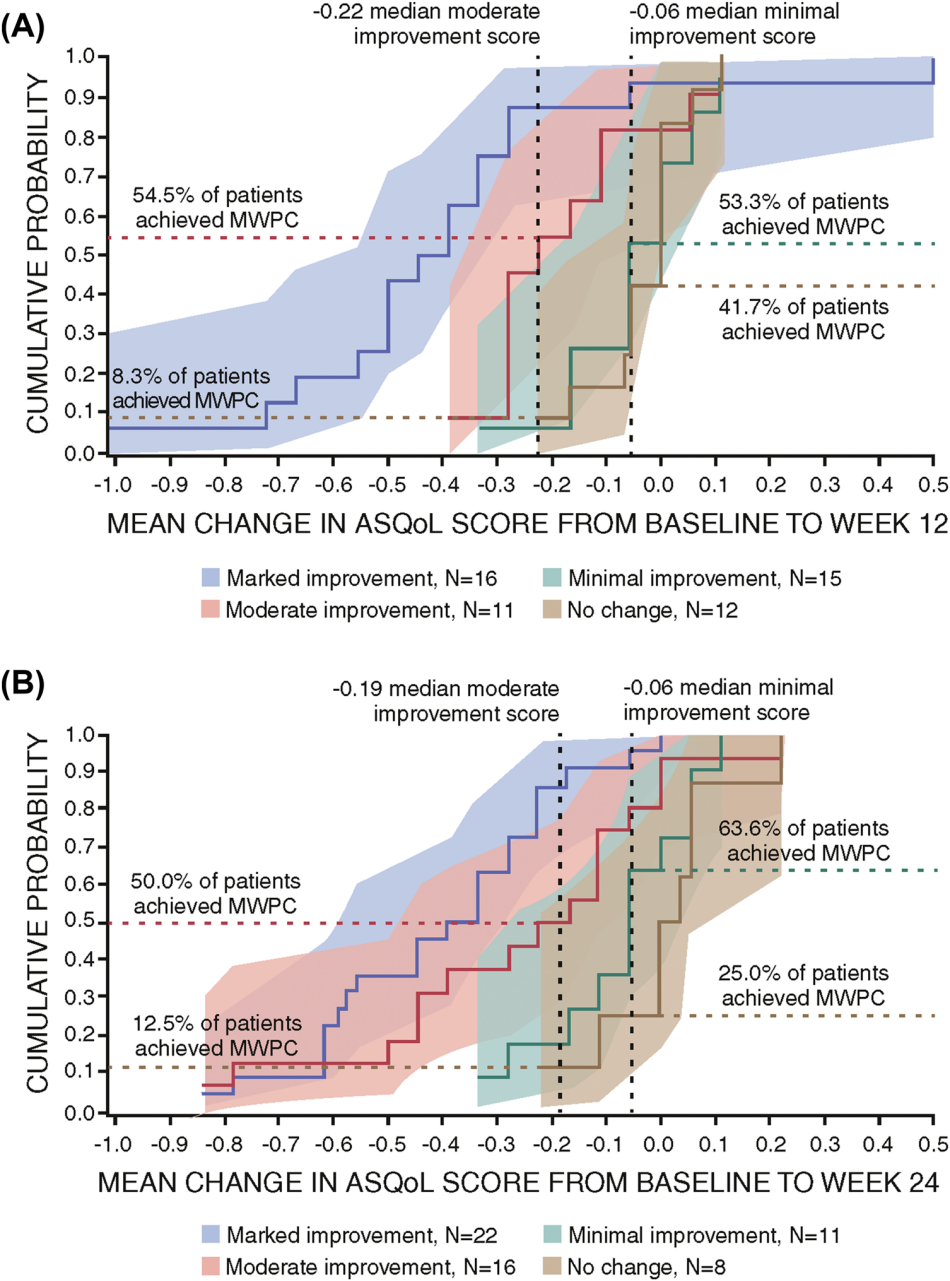
Cumulative distribution function of change in ASQoL score from Baseline to Week 12 (**a**) and to Week 24 (**b**) stratified by patient global impression of change (PGIC) anchor groups indexed against median ASQoL change score for minimal and moderate improvement groups in the nr-axSpA with objective signs of inflammation subgroup

The eCDF for change in ASQoL score stratified by PGIC anchor groups is presented in Fig. 3. The cumulative percentage of patients with PGIC-based moderate improvement versus those with no change meeting or exceeding the MWPC point estimate (4-point improvement, ASQoL sum score) were 54.5 and 8.3%, respectively, at Week 12 (a 46.2 percentage point advantage). At Week 24 these same cumulative percentages were 50.0 and 12.5%, respectively (a 37.5 percentage point advantage). This evidence supports the use of the 4-point improvement estimate for clinically meaningful change within this population.

## Discussion

This study aimed to assess the psychometric properties of the ASQoL in patients with nr-axSpA. We aimed to evaluate the existence and nature of domains in the ASQoL and their relative contribution to score precision relative to the total score to assess acceptable reliability, validity, and ability to detect change, including clinically meaningful change estimates of the ASQoL in this patient population. The primary motivation of this exercise was to confirm the absence of any meaningful departure from known psychometric properties of the ASQoL within this population.

The ASQoL was chosen as the most appropriate PRO for the assessment of quality of life at the time the Phase III study was conducted, from which the patient data in this analysis were derived; the ASAS Health Index [47], an AS-specific tool assessing overall patient functioning and health, had not been developed at that time. Compared to other PRO measures, the ASQoL has been extensively studied in SpA for hypothesis testing and reliability [48].

This high endorsement for all but two items of the ASQoL as seen from the response pattern evaluation suggests that an additional response category on the high end of the response scale might increase sensitivity.

There are several methodological features to consider when evaluating these results. Firstly, the estimation of highly parameterized models generally requires large sample sizes to achieve acceptable parameter estimate precision. In the case of latent variable models, like IRT, models are generally highly parameterized. In the case of the models fit to the ASQoL in this study, this was not the case, and the number of estimated parameters across models ranged from 36 to 54 in a sample of *n* = 325. There is a legitimate concern as to whether under such circumstances parameters are estimated with acceptable stability. Though evidence on the finite sample performance of these models is limited, in the comprehensive simulation study conducted by Forero and Maydeu-Olivares [49], item parameter relative bias for the types of IRT models fit in this trial never exceeded 6% across any of the simulation conditions examined. Therefore, we believe the pooled sample item parameter estimates were not systematically biased for these models estimated in a sample of *n* = 325.

While the finite sample was not expected to result in estimation error for the pooled sample given the simulation evidence reported by Forero and Maydeus-Olivares [49], the same cannot be said of the DIF analysis. Because this required stratified item parameter estimation, the item parameters for the nr-axSpA population were based on a very limited sample size. The procedure used to detect DIF has a known inflated type I error rate. Given these issues, we anticipated over-detection of DIF. To address this, wABC statistics were used to graphically evaluate the severity of any detected DIF; however, none was detected. Ultimately, there is little evidence to suggest that different means of diagnosing the condition will yield differential item bias. However, DIF analysis in such small samples must be conducted with extreme caution and consequently interpreted with substantial caution. This is a limitation of all work in rare disease populations.

The use of test–retest reliability in interventional designs where the retest interval spans the interventional period is inconsistent with the premise of test–retest reliability. Under classical definitions, the retest interval is to only contain an effect of time. No material intervention that could alter responses should intervene in the time effect. The solution employed in the regulatory space is to identify a subgroup reporting no change in the retest interval on an external anchor variable, which is the approach followed here. And yet, it is at minimum tautological to report the degree of reproducibility of scores among people reporting no change, and then simultaneously odd that the ICC(2,1) is not 1 in this subgroup. This evidence legitimately calls into question the validity of the anchor variables and the accuracy with which this approach is capable of characterizing test–retest reliability or long-term stability, for that matter. Fortunately, the test–retest reliability of the ASQoL has been established previously in a non-interventional context, and we make no claim of issue with the ASQoL. The anchor-based approach in the regulatory environment is legitimately questioned.

In terms of study limitations, the ASQoL total score is derived from a mix of symptoms and impacts, which is generally discouraged by regulatory guidance [50]. However, the mix of symptoms and impacts in the ASQoL domains are inherently logical and the ASQoL scores demonstrated robust psychometric properties. Given the small size of the patient subgroup who had nr-axSpA with objective signs of inflammation, it was difficult to draw firm conclusions about the likelihood of separation of treatment arms at the meaningful change location. The overlap in confidence band width was de-emphasized over the detected differences in cumulative proportions and separation of eCDFs; however, with a larger number of patients, the confidence band width would be expected to shrink resulting in significant separation at these meaningful change locations.

In conclusion, our findings provide the first evidence supporting the use of the ASQoL as an outcome measure for use in future clinical trials involving patients with nr-axSpA. However, further studies in a larger nr-axSpA cohort are needed to validate its suitability.

## Data sharing statement

Due to the small size of the patient subgroup with nr-axSpA and objective signs of inflammation, Individual Patient Data cannot be adequately anonymized and there is a reasonable likelihood that individual participants could be re-identified. For this reason, data from this study cannot be shared.

## Electronic supplementary material

Below is the link to the electronic supplementary material.

Supplementary file1 (DOCX 379 kb)
