# Supplementary material for: Validation of the Ankylosing Spondylitis Quality of Life assessment tool in patients with non-radiographic axial spondyloarthritis
Source: Qual Life Res. 2020 Oct 31;30(3):945–54. doi: 10.1007/s11136-020-02686-0 (PMC7952291; doi:10.1007/s11136-020-02686-0)
Supplement: Supplementary file 1 — Supplementary file1 (DOCX 379 kb) [file 11136_2020_2686_MOESM1_ESM.docx]

***Supplementary Appendix – IRT model selection and DIF results***

**Validation of the Ankylosing Spondylitis Quality of Life assessment tool in patients with non-radiographic axial spondyloarthritis**

*Bengt Höpken, Daniel Serrano, Kristina Harris, Mark Hwang, John Reveille*

**Contents**

[List of abbreviations 2](#_Toc51335623)

[IRT model selection 3](#_Toc51335624)

[Table S1. C2-based IRT Model Fit Assessments 4](#_Toc51335625)

[Table S2. Marginal fit statistics for four IRT models considered 5](#_Toc51335626)

[Figure S1. Baseline bifactor model local dependence assessment 6](#_Toc51335627)

[Figure S2. Baseline MIRT model local dependence assessment 7](#_Toc51335628)

[Figure S3. Baseline unidimensional 2PL IRT model local dependence assessment 8](#_Toc51335629)

[Figure S4. Baseline unidimensional Rasch model (ASQoL development model) local dependence assessment 9](#_Toc51335630)

[IRT item parameters 10](#_Toc51335631)

[Table S3. MIRT and bifactor slopes 11](#_Toc51335632)

[Table S4. Model parameters 12](#_Toc51335633)

[DIF 13](#_Toc51335634)

[Table S5. DIF between the mNY-AS (n=178) and mNY-nr-axSpA (n=147) definitions based upon the Wald-2 χ2 sweep procedure 14](#_Toc51335635)

[Table S6. DIF between the OSI-axSpA (n=258) and OSI-nr-axSpA (n=67) definitions based upon the Wald-2 χ2 sweep procedure 15](#_Toc51335636)

[DIF severity assessment 16](#_Toc51335637)

[Figure S5. DIF Severity for ASQoL Item 7, "Always Fatigued" 17](#_Toc51335638)

[Figure S6. DIF Severity for ASQoL Item 12, "Easily Fatigued" 18](#_Toc51335639)

## List of abbreviations

| 2PL | Two-parameter logistic model |
| --- | --- |
| AS | Ankylosing spondylitis |
| ASQoL | Ankylosing Spondylitis Quality of Life |
| axSpA | Axial spondyloarthritis |
| DIF | Differential item functioning |
| IRT | Item response theory |
| LD | Local dependence |
| MIRT | Multidimensional item response theory |
| mNY | Modified New York |
| nr-axSpA | Non-radiographic axial spondyloarthritis |
| OSI | Observed signs of inflammation |
| RMSEA | Root mean squared error of approximation |
| SPARCC | Spondyloarthritis Research Consortium of Canada |
| wABC | Weighted area between curves |

## IRT model selection

Model fit indices for the considered IRT models estimated at baseline are presented in **Table S1**. Four models were considered for the 18 binary ASQoL items. In order, these models were a unidimensional Rasch model (slopes constrained to equality across items), the 2PL IRT extension of the Rasch model (slopes freely estimated for each item), a 4-factor MIRT (a multidimensional extension of the 2PL, and may be thought of as a 4-factor CFA), and the bifactor extension of the MIRT (the 4-factor CFA to which a general domain was added upon which all items loaded). While the fit indices for IRT models considered were comparable in information criteria, only the bifactor model satisfied the test of exact fit as demonstrated in the 90% confidence interval for the C2-based RMSEA having a lower bound of 0 and an upper bound <0.05. Consequently, model fit unambiguously favored the bifactor model as the optimal model within which to assess item properties. The model fit criterion was part one of three criteria used to identify the optimal model. The remaining two criteria examined to finalize the optimal model determination included local dependence and stability of IRT slopes.

## Table S1. C2-based IRT Model Fit Assessments

| **FIT STATISTIC** | **RASCH** | **UNI-IRT** | **4-FACTOR MIRT** | **BIFACTOR** |
| --- | --- | --- | --- | --- |
| # of Estimated Parameters | 19 | 36 | 42 | 54 |
| -2LL | 5899.66 | 5849.12 | 5731.0 | 5697.1 |
| AIC | 5937.66 | 5921.12 | 5815.0 | 5805.1 |
| BIC | 6009.62 | 6057.45 | 5974.05 | 6009.6 |
| C2 χ2 | 407.12 | 373.5 | 203.53 | 160.65 |
| C2 DF | 152 | 135 | 129 | 117 |
| C2 p-value | 0.0001 | 0.0001 | 0.0001 | 0.0046 |
| C2 RMSEA | 0.07 | 0.07 | 0.04 | 0.03 |
| C2 RMSEA 90% CI | 0.046, 0.094 | 0.053, 0.088 | 0.017, 0.059 | 0.000, 0.049 |

**Interpretation of fit indices:**

- - - - 1. C2 χ2: Non-significant p indicates perfect fit, test is overpowered, thus small samples enable achievement of this criterion.
        2. AIC: Smaller values indicate better fit
        3. BIC: Smaller values indicate better fit
        4. RMSEA: Values less than 0.1 indicate acceptable fit, values less than 0.05 indicate strong fit, values of 0 indicate perfect fit.
        5. RMSEA 90% CI: lower limit of 0 with upper limit < 0.05 indicate perfect fit

The highlighted column denotes the best fitting model

-2LL: -2 times the log likelihood; AIC: Akaike’s information criterion; BIC: Bayesian information criterion; C2 limited information fit approximation to the G2 χ2 statistic for sparse contingency tables (currently the most precise fit index); CI: confidence interval; DF: degrees of freedom; IRT: item response theory; MIRT: multidimensional item response theory; RMSEA: root mean squared error of approximation; χ2: Chi-square test.

The four models were compared on the marginal χ2 fit index for each item. Values of zero indicate appropriate alignment between observed and expected values. All models achieved this criterion, with only trivial deviations observed for the 2PL and MIRT models for only items ASQoL 6 and ASQoL 11 (**Table S2**). Thus, the models were not differentiable on this criterion.

## Table S2. Marginal fit statistics for four IRT models considered

| **ITEM** | **RASCH** | **2PL** | **MIRT** | **BI- FACTOR** |
| --- | --- | --- | --- | --- |
| ASQOL 1 | 0 | 0 | 0 | 0 |
| ASQOL 2 | 0 | 0 | 0 | 0 |
| ASQOL 3 | 0 | 0 | 0 | 0 |
| ASQOL 4 | 0 | 0 | 0 | 0 |
| ASQOL 5 | 0 | 0 | 0 | 0 |
| ASQOL 6 | 0 | 0.1 | 0 | 0 |
| ASQOL 7 | 0 | 0 | 0 | 0 |
| ASQOL 8 | 0 | 0 | 0 | 0 |
| ASQOL 9 | 0 | 0 | 0 | 0 |
| ASQOL 10 | 0 | 0 | 0 | 0 |
| ASQOL 11 | 0 | 0.1 | 0.1 | 0 |
| ASQOL 12 | 0 | 0 | 0 | 0 |
| ASQOL 13 | 0 | 0 | 0 | 0 |
| ASQOL 14 | 0 | 0 | 0 | 0 |
| ASQOL 15 | 0 | 0 | 0 | 0 |
| ASQOL 16 | 0 | 0 | 0 | 0 |
| ASQOL 17 | 0 | 0 | 0 | 0 |
| ASQOL 18 | 0 | 0 | 0 | 0 |

2PL: two-parameter logistic model; IRT: item response theory; MIRT: multidimensional item response theory.

Chen’s standardized LD X2 statistics are presented as heatmaps for all items for each model in **Figures S1, S2, S3** and **S4**. In all figures, darker colors indicate stronger local dependence between the items, with values exceeding 3, indicating potentially serious local dependence. While the 4-factor MIRT solution minimized these statistics, the bifactor model was a close second, with only one large LD estimate detected between ASQoL 13 (frustrated) and ASQoL 7 (always fatigued). Both unidimensional models were contaminated with persistent LD. Given the model fit findings and reasonable achievement of conditional independence, the bifactor model remained favored, but MIRT domains demonstrated value in shrinking LD. Item parameters were examined next to determine whether models differed in the stability of IRT slopes. Slopes were examined because they are the least biased item reliabilities.

## Figure S1. Baseline bifactor model local dependence assessment


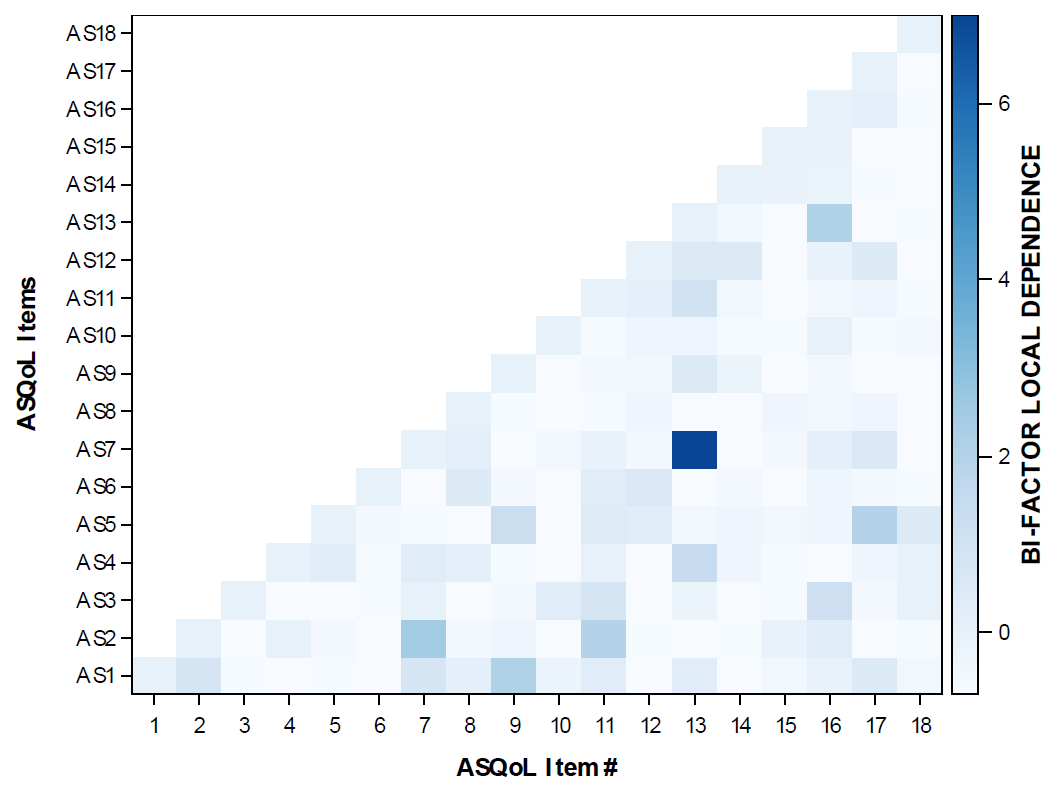


## Figure S2. Baseline MIRT model local dependence assessment


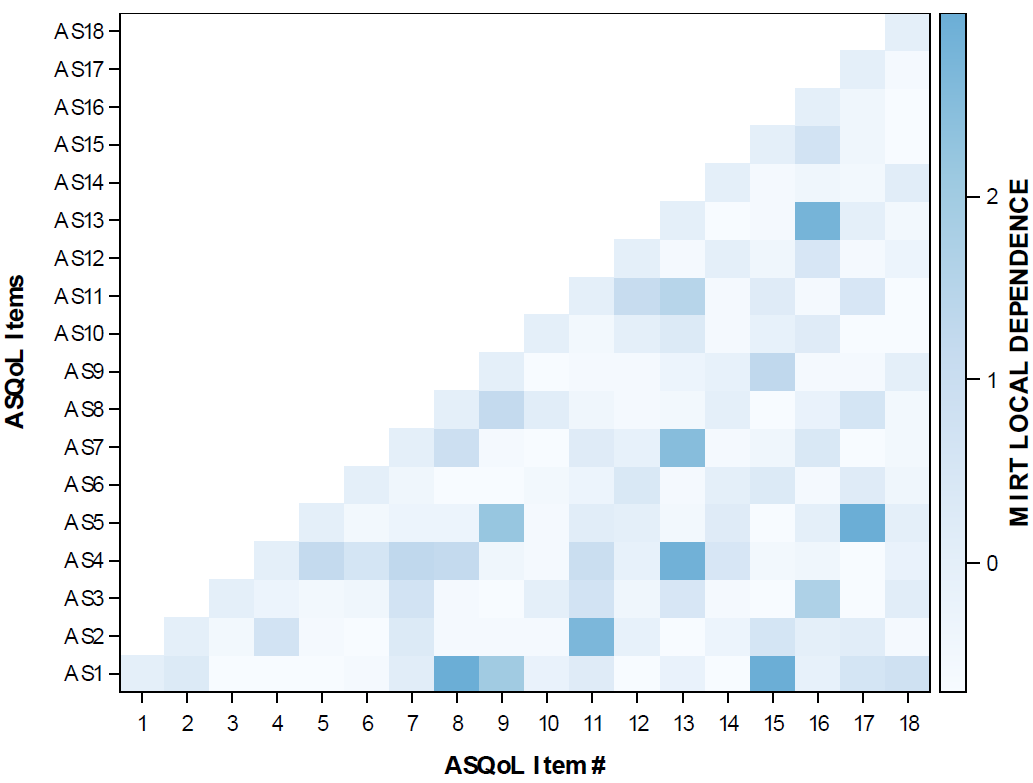


MIRT: multidimensional item response theory.

## Figure S3. Baseline unidimensional 2PL IRT model local dependence assessment


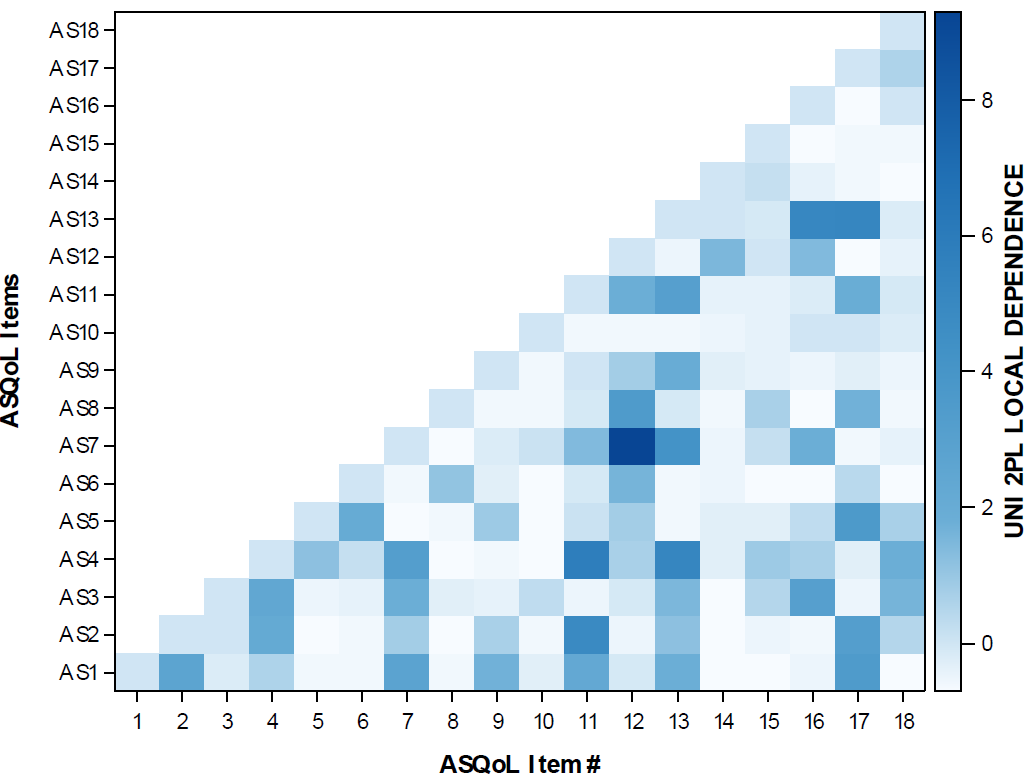


2PL: two-parameter logistic model; IRT: item response theory.

## Figure S4. Baseline unidimensional Rasch model (ASQoL development model) local dependence assessment


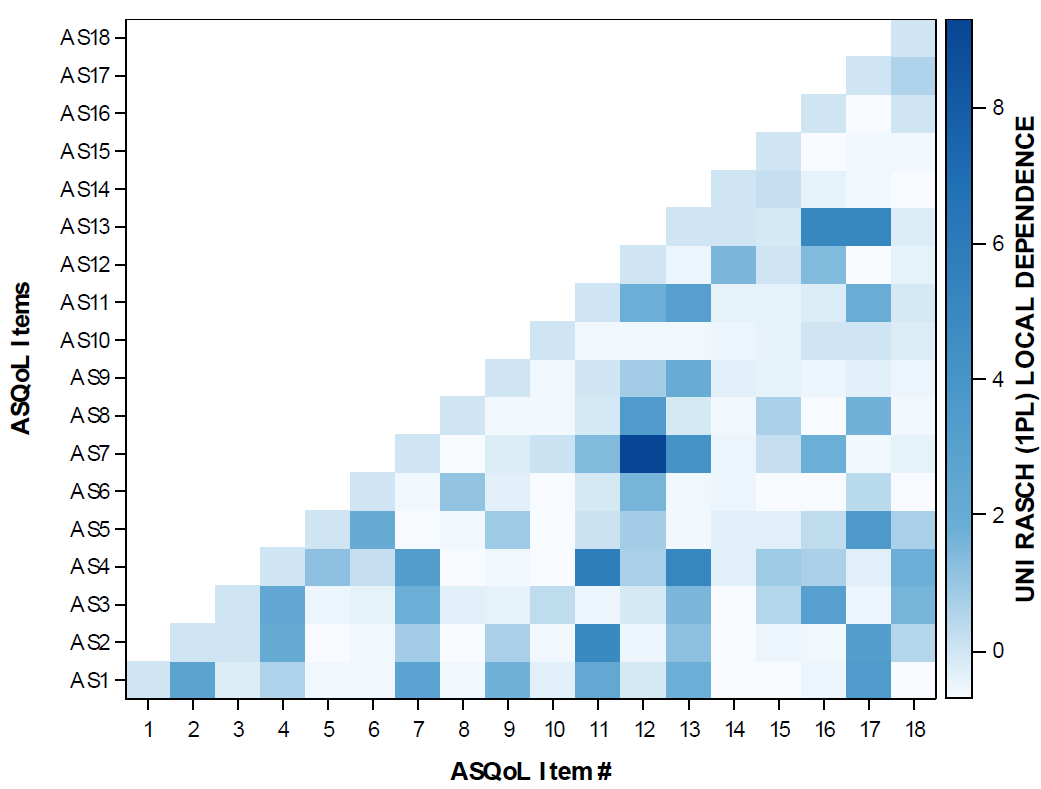


1PL: one-parameter logistic model.

## IRT item parameters

As seen in **Table S3**, all slopes were estimated within a reasonable range (values between 0.7 and 3), with the exception of ASQoL 4 (struggle to do chores) and ASQoL 12 (easily fatigued), whose slopes exceeded 3 and crept toward or above 5 for both models. Considering the slope stability findings in the context of the model fit and LD results, the bifactor was selected as the final model from which test and score properties would be evaluated. DIF assessments of this solution are presented to evaluate the extent to which this solution generalized across the mNY-nr-axSpA and mNY-AS populations and separately across the OSI-nr-axSpA and OSI-axSpA populations.

## Table S3. MIRT and bifactor slopes

| **ITEMS** | **STEM** | **FACTORS** | **MML MIRT SLOPE** | **MML BIFACTOR SLOPE** |
| --- | --- | --- | --- | --- |
| ASQOL 1 | Limits | ADLS | 1.95 | 1.80 |
| ASQOL 3 | Dressing | ADLS | 1.21 | 0.92 |
| ASQOL 4 | Struggle chores | ADLS | 3.71 | 5.22 |
| ASQOL 9 | Unbearable pain | ADLS | 1.67 | 1.61 |
| ASQOL 10 | Morning delay | ADLS | 1.45 | 1.43 |
| ASQOL 11 | Unable chores | ADLS | 2.77 | 2.32 |
| ASQOL 14 | Always pain | ADLS | 1.15 | 1.17 |
| ASQOL 16 | Hair | ADLS | 1.39 | 1.16 |
| ASQOL 5 | Sleep disturbance | INSOMNIA | 1.14 | 1.10 |
| ASQOL 6 | Unable activities | INSOMNIA | 2.81 | 2.08 |
| ASQOL 7 | Always fatigued | FATIGUE | 2.32 | 1.79 |
| ASQOL 8 | Rest | FATIGUE | 2.07 | 1.91 |
| ASQOL 12 | Easily fatigued | FATIGUE | 4.64 | 5.53 |
| ASQOL 2 | Crying | EMOTION | 1.40 | 1.15 |
| ASQOL 13 | Frustrated | EMOTION | 2.31 | 1.88 |
| ASQOL 15 | Miss out | EMOTION | 2.47 | 2.51 |
| ASQOL 17 | Depressed | EMOTION | 1.83 | 1.73 |
| ASQOL 18 | Disappoint | EMOTION | 1.59 | 1.45 |

ASQoL 4 and 12 are highlighted because the slopes were approaching upper bounds for stability of slopes. ADLS: activities of daily living; ASQoL: Ankylosing Spondylitis Quality of Life; MIRT: multidimensional item response theory; MML: marginal maximum likelihood.

## Table S4. Model parameters

|  |  |  | **Bifactor Slopes** | | | | | |
| --- | --- | --- | --- | --- | --- | --- | --- | --- |
| **ITEMS** | **STEM** | **FACTORS** | **General Slope** | **R1 Slope** | **R2 Slope** | **R3 Slope** | **R4 Slope** | **Intercept** |
| ASQOL 1 | Limits | ADLS | 1.80 | 0.76 | 0.00 | 0.00 | 0.00 | 1.37 |
| ASQOL 3 | Dressing | ADLS | 0.92 | 0.85 | 0.00 | 0.00 | 0.00 | 0.61 |
| ASQOL 4 | Struggle chores | ADLS | 5.22 | 4.72 | 0.00 | 0.00 | 0.00 | 0.65 |
| ASQOL 9 | Unbearable pain | ADLS | 1.61 | 0.51 | 0.00 | 0.00 | 0.00 | 4.61 |
| ASQOL 10 | Morning delay | ADLS | 1.43 | 0.40 | 0.00 | 0.00 | 0.00 | 0.52 |
| ASQOL 11 | Unable chores | ADLS | 2.32 | 1.53 | 0.00 | 0.00 | 0.00 | 0.38 |
| ASQOL 14 | Always pain | ADLS | 1.17 | 0.15 | 0.00 | 0.00 | 0.00 | 1.33 |
| ASQOL 16 | Hair | ADLS | 1.16 | 0.93 | 0.00 | 0.00 | 0.00 | 1.84 |
| ASQOL 5 | Sleep disturbance | INSOMNIA | 1.10 | 0.00 | 0.88 | 0.00 | 0.00 | 1.22 |
| ASQOL 6 | Unable activities | INSOMNIA | 2.08 | 0.00 | 0.82 | 0.00 | 0.00 | 2.88 |
| ASQOL 7 | Always fatigued | FATIGUE | 1.79 | 0.00 | 0.00 | 1.42 | 0.00 | -1.03 |
| ASQOL 8 | Rest | FATIGUE | 1.91 | 0.00 | 0.00 | 0.81 | 0.00 | 8.57 |
| ASQOL 12 | Easily fatigued | FATIGUE | 5.53 | 0.00 | 0.00 | 5.76 | 0.00 | 1.39 |
| ASQOL 2 | Crying | EMOTION | 1.15 | 0.00 | 0.00 | 0.00 | 0.94 | 1.99 |
| ASQOL 13 | Frustrated | EMOTION | 1.88 | 0.00 | 0.00 | 0.00 | 1.35 | 1.59 |
| ASQOL 15 | Miss out | EMOTION | 2.51 | 0.00 | 0.00 | 0.00 | 0.60 | -1.75 |
| ASQOL 17 | Depressed | EMOTION | 1.73 | 0.00 | 0.00 | 0.00 | 1.99 | 1.64 |
| ASQOL 18 | Disappoint | EMOTION | 1.45 | 0.00 | 0.00 | 0.00 | 0.68 | 0.62 |

Estimated 54 item parameters on n=325 subjects. ADLS: activities of daily living; MML: marginal maximum likelihood; MIRT: multidimensional item response theory.

## DIF

DIF was tested to determine whether the final ASQoL solution obtained in the pooled axSpA population functioned identically between the AS/axSpA and nr-axSpA populations. The DIF was tested on the primary dimension of the bifactor solution presented in **Table S3**. Two definitions for AS/axSpA and nr-axSpA were considered: (1) the definition based upon mNY+ which yielded n=178 mNY-AS subjects and n=147 mNY‑nr‑axSpA subjects; (2) the OSI definition, a more stringent definition based upon both no radiographic evidence and clear inflammatory biomarkers (C-reactive protein>upper limit of normal and/or SPARCC≥2), which yielded n=258 OSI-axSpA subjects and n=67 OSI-nr-axSpA subjects.

The first assessment of DIF based upon the mNY+ definition is presented in **Table S5**. As seen there, ASQoL 7 (‘always fatigued’) and ASQoL 12 (‘easily fatigued’) demonstrated significant DIF based upon the Wald-2 sweep procedure. These items were examined to assess whether this significant DIF was meaningful or not via DIF severity effect size estimates. It was not meaningful, as described in the Differential item functioning severity assessment section. In addition, using the more stringent OSI definition, no items demonstrated significant DIF between the OSI-nr-axSpA and OSI-axSpA, though the smaller sample size (n=67) of the OSI-nr-axSpA population likely increased the dispersion, thereby attenuating detection of the DIF effect in ASQoL 7 and ASQoL 12 (see **Table S6**).

## Table S5. DIF between the mNY-AS (n=178) and mNY-nr-axSpA (n=147) definitions based upon the Wald-2 χ2 sweep procedure

| **OMNIBUS** | | | | **SLOPE** | | **INTERCEPT** | |
| --- | --- | --- | --- | --- | --- | --- | --- |
| **ITEM** | **ITEM STEM** | **χ2** | **P** | **χ2** | **P** | **χ2** | **P** |
| AS1 | Limits | 0 | 1 | 0 | 1 | 0 | 1 |
| AS2 | Crying | 0 | 1 | 0 | 1 | 0 | 1 |
| AS3 | Dressing | 0 | 1 | 0 | 1 | 0 | 1 |
| AS4 | Struggle chores | 0 | 1 | 0 | 1 | 0 | 1 |
| AS5 | Sleep disturbance | 0 | 1 | 0 | 1 | 0 | 1 |
| AS6 | Unable activities | 0 | 1 | 0 | 1 | 0 | 1 |
| AS7 | Always fatigued | 9.9 | 0.0196 | 1.3 | 0.5296 | 8.6 | 0.0034 |
| AS8 | Rest | 0 | 1 | 0 | 1 | 0 | 1 |
| AS9 | Unbearable pain | 0 | 1 | 0 | 1 | 0 | 1 |
| AS10 | Morning delay | 0 | 1 | 0 | 1 | 0 | 1 |
| AS11 | Unable chores | 0 | 1 | 0 | 1 | 0 | 1 |
| AS12 | Easily fatigued | 8.6 | 0.0347 | 0.1 | 0.9344 | 8.5 | 0.0036 |
| AS13 | Frustrated | 0 | 1 | 0 | 1 | 0 | 1 |
| AS14 | Always pain | 0 | 1 | 0 | 1 | 0 | 1 |
| AS15 | Miss out | 0 | 1 | 0 | 1 | 0 | 1 |
| AS16 | Hair | 0 | 1 | 0 | 1 | 0 | 1 |
| AS17 | Depressed | 0 | 1 | 0 | 1 | 0 | 1 |
| AS18 | Disappoint | 0 | 1 | 0 | 1 | 0 | 1 |

Items AS7 and AS12 demonstrated significant DIF between the mNY+ and mNY- definitions for AS and nr-axSpA based upon the Wald-2 χ2 sweep procedure. This definition yielded n=147 subjects with mNY-nr-axSpA and n=178 with mNY-AS. DIF was restricted to the intercept and did not affect the slope. However, the DIF severity effect size demonstrated that this significant DIF was not meaningful, therefore no meaningful DIF existed in the ASQoL and thus the ASQoL functioned identically between mNY-nr-axSpA and mNY-AS. AS: ankylosing spondylitis; mNY: modified New York; nr-axSpA: non-radiographic axial spondyloarthritis; χ2: Chi-square test.

## Table S6. DIF between the OSI-axSpA (n=258) and OSI-nr-axSpA (n=67) definitions based upon the Wald-2 χ2 sweep procedure

|  | | **OMNIBUS** | | **SLOPE** | | **INTERCEPT** | |
| --- | --- | --- | --- | --- | --- | --- | --- |
| **ITEM** | **ITEM STEM** | **χ2** | **P** | **χ2** | **P** | **χ2** | **P** |
| AS1 | Limits | 1.1 | 0.7787 | 0.9 | 0.6377 | 0.2 | 0.6604 |
| AS2 | Crying | 3.4 | 0.3291 | 2.4 | 0.2973 | 1 | 0.3151 |
| AS3 | Dressing | 1.2 | 0.7605 | 0 | 0.9784 | 1.1 | 0.2892 |
| AS4 | Struggle chores | 0.4 | 0.9355 | 0.2 | 0.9191 | 0.3 | 0.6146 |
| AS5 | Sleep disturbance | 1.8 | 0.6252 | 0.2 | 0.9046 | 1.6 | 0.2132 |
| AS6 | Unable activities | 0.3 | 0.9521 | 0.2 | 0.8847 | 0.1 | 0.7567 |
| AS7 | Always fatigued | 3 | 0.3952 | 0.2 | 0.899 | 2.8 | 0.097 |
| AS8 | Rest | 4 | 0.2598 | 0.7 | 0.7016 | 3.3 | 0.0685 |
| AS9 | Unbearable pain | 6.1 | 0.1081 | 3.3 | 0.1951 | 2.8 | 0.096 |
| AS10 | Morning delay | 1 | 0.7961 | 0.3 | 0.8516 | 0.7 | 0.4031 |
| AS11 | Unable chores | 0.2 | 0.9764 | 0 | 0.9887 | 0.2 | 0.6675 |
| AS12 | Easily fatigued | 3.2 | 0.3651 | 1.7 | 0.4355 | 1.5 | 0.2187 |
| AS13 | Frustrated | 1.9 | 0.5963 | 1 | 0.6077 | 0.9 | 0.3458 |
| AS14 | Always pain | 3.9 | 0.278 | 2.6 | 0.2775 | 1.3 | 0.2568 |
| AS15 | Miss out | 1.4 | 0.6978 | 0 | 0.9811 | 1.4 | 0.2381 |
| AS16 | Hair | 2.2 | 0.5404 | 1.7 | 0.4298 | 0.5 | 0.4942 |
| AS17 | Depressed | 2.8 | 0.4241 | 1.8 | 0.4083 | 1 | 0.3165 |
| AS18 | Disappoint | 1.5 | 0.675 | 1.1 | 0.5828 | 0.5 | 0.5018 |

No items demonstrated significant DIF between the OSI definition of axSpA and nr-axSpA based upon the Wald-2 χ2 sweep procedure. This definition yielded n=67 subjects with nr-axSpA and n=258 with axSpA. Therefore, no meaningful DIF existed in the ASQoL and thus the ASQoL functioned identically between OSI-nr-axSpA and OSI-axSpA. AS: ankylosing spondylitis; mNY: modified New York; nr-axSpA: non-radiographic axial spondyloarthritis; χ2: Chi-square test.

## DIF severity assessment

No significant DIF was detected between the OSI-nr-axSpA and OSI-axSpA population. However, significant DIF was detected for two items between the mNY-nr-axSpA and mNY-AS populations. These items were examined for DIF severity between the mNY-nr-axSpA and mNY-AS populations. Examination of the DIF associated with ASQoL 7 (“always fatigued”) and ASQoL 12 (“easily fatigued”) using the weighted area between curves (wABC) DIF severity index revealed no meaningful DIF between the mNY-nr-axSpA and mNY-AS populations. The common cut-off for the wABC is 0.3 or greater. The DIF for ASQoL 7 and ASQoL 12 was associated with wABC estimates of 0.20 and 0.11, respectively. These DIF severity estimates are characterized in **Figure S5** and **Figure S6**. While the response probability curves differ between the populations, they do so in the extreme low end of the normal distribution (X-axis values below -2 on a Z-score distribution), where virtually 0% of the sample would exist, which accounts for the low wABC estimates. Therefore, the significant DIF arose from false-positive detection as demonstrated by the wABC. Consequently, no meaningful DIF was detected between the AS/axSpA and nr-axSpA populations regardless of whether the mNY or OSI definitions were employed, demonstrating that the psychometric properties of the ASQoL are uniform irrespective of the form of axSpA. Given the DIF evidence, scoring and Classical Test Theory (CTT) evaluations of scores, as well as the assessment of sensitivity to detect change and meaningful change were computed in the OSI-nr-axSpA population.

## Figure S5. DIF Severity for ASQoL Item 7, "Always Fatigued"


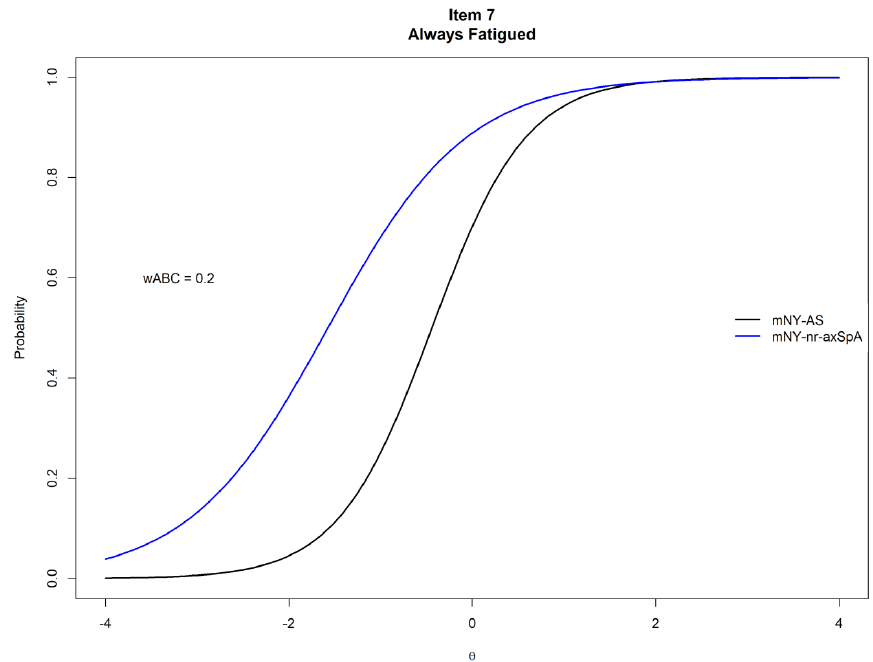


AS: ankylosing spondylitis; DIF: differential item functioning; mNY: modified New York; nr-axSpA: non-radiographic axial spondyloarthritis; wABC: weighted area between curves.

## Figure S6. DIF Severity for ASQoL Item 12, "Easily Fatigued"


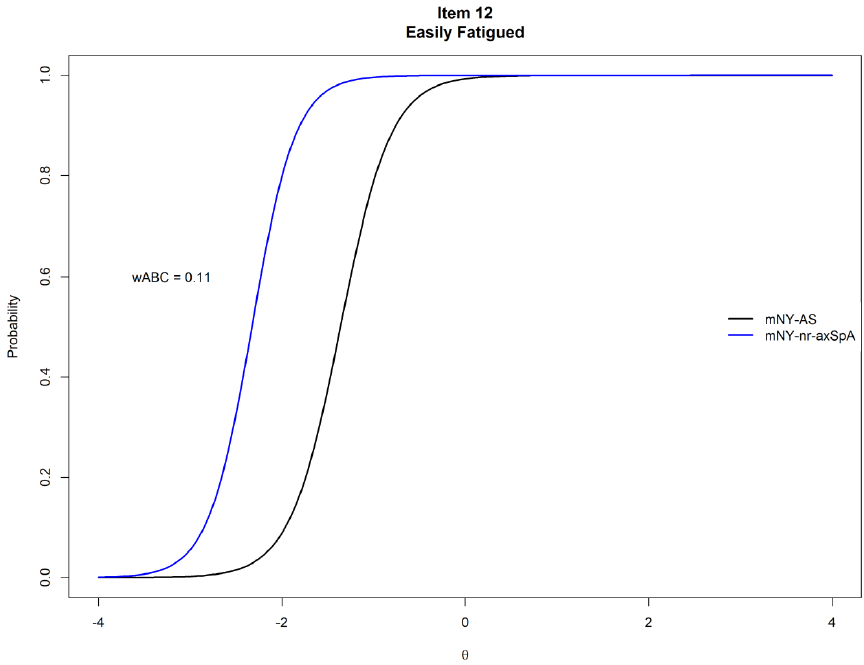


AS: ankylosing spondylitis; DIF: differential item functioning; mNY: modified New York; nr-axSpA: non-radiographic axial spondyloarthritis; wABC: weighted area between curves.
